# Mint (*Mentha* spp.) Honey: Analysis of the Phenolic Profile and Antioxidant Activity

**DOI:** 10.17113/ftb.60.04.22.7703

**Published:** 2022-12

**Authors:** Tomislav Pavlešić, Sanja Poljak, Dijana Mišetić Ostojić, Ivana Lučin, Christian A. Reynolds, Daniela Kalafatovic, Lara Saftić Martinović

**Affiliations:** 1 University of Rijeka, Faculty of Health Studies, Viktora Cara Emina 5, 51000 Rijeka, Croatia; 2 University of Rijeka, Trg braće Mažuranića 10, 51000 Rijeka, Croatia; 3 University of Rijeka, Department of Biotechnology, Radmile Matejčić 2, 51000 Rijeka, Croatia; 4 Croatian Veterinary Institute, Veterinary Institute Rijeka, Laboratory for Analytical Chemistry and Residues, Podmurvice 29, 51000 Rijeka; 5 University of Rijeka, Faculty of Engineering, Vukovarska 58, 51000 Rijeka, Croatia; 6 Center for Advanced Computing and Modelling, University of Rijeka, Radmile Matejčić 2, 51000 Rijeka, Croatia

**Keywords:** mint (*Mentha* spp.) honey, honey chemical characterization, physicochemical properties, melissopalynology, food authentication

## Abstract

**Research background:**

The composition of honey is influenced by the botanical source and geographical area of the nectar from which it is derived. Unifloral honeys reach higher market value than multifloral honeys due to their specific aromas, which result from volatile and phenolic compounds.

**Experimental approach:**

The aim of our study is to characterize the phenolic composition of a rare unifloral variety of honey – mint (*Mentha* spp.) honey. For this purpose, we performed standard physicochemical analyses, pollen analysis, determined total phenolic and flavonoid content, analysed antioxidant activity and performed qualitative and quantitative analyses of phenolic compounds for five mint honeys.

**Results and conclusions:**

Our results indicate that mint honey samples have high phenolic content, expressed in gallic acid equivalents, from (76.7±0.6) to (90.1±1.1) mg/100 g, and flavonoid content, expressed as quercetin equivalents, from (6.7±0.6) to (12.5±0.8) mg/100 g. These honey samples also exhibit strong antioxidant activity, expressed as Trolox equivalents, from (33.6±2.8) to (51.3±1.2) mg/100 g and from (14.4±0.8) to (55.1±2.4) mg/100 g when analysed with DPPH and ABTS assays, respectively. Quantitative LC-MS/MS analysis revealed that the most abundant phenols in all samples were chrysin, apigenin and *p*-coumaric acid. Qualitative LC-MS/MS analysis identified the presence of kaempferide, diosmetin, acacetin and several caffeic acid derivatives.

**Novelty and scientific contribution:**

Our study indicates that mint honey contains unique phenolic profiles, which likely contribute to its distinctive aroma and strong antioxidant activity. A detailed description of the rare honey varieties gives beekeepers greater visibility and easier access to the demanding natural product market.

## INTRODUCTION

Honey is a natural food, consisting mainly of sugars, enzymes, amino acids, organic acids, vitamins, minerals and aromatic substances. As people increasingly consume honey products, standards and norms that guarantee its identity and quality are needed (*1*). According to the European Union Directive 2001/110/EC (*2*), honey is defined as a sweet, thick, viscous, liquid or crystallized product produced by honey bees from nectar plants, secretion of living plant parts or secretions of sucking insects.

The composition of honey is influenced by the botanical origin and the geographical area of the nectar from which it is obtained (*3*). Unifloral honeys reach higher market values ​​than their multifloral counterparts. The reason for this is the specific aroma profiles of unifloral honeys, which result from their unique composition of volatile compounds. In addition to specific organoleptic properties, honey has other components that contribute to its nutritional and medicinal value. Proteins, vitamins, minerals, organic acids and phenolic compounds, the most variable components of honey, contribute predominantly to the strong bioactive effects of this natural food. Therefore, honey is appreciated by consumers, not only in the food industry, but also in the pharmaceutical and cosmetic industries.

*Mentha* L. is a nectar-producing plant that has about 25 species and hybrids (*4*). It belongs to the family *Lamiaceae*, subfamily *Nepetoideae*, tribe *Menthae*, which are predominantly characterized as aromatic plants. Due to the presence of numerous aromatic constituents, *Mentha* spp. essential oils are widely used. Over nine different chemotypes of mint essential oil have been reported (*4*). Mint leaves have a unique composition of volatile compounds and contain many bioactive phenolic compounds, which is why they are widely used medicinally, especially for the treatment of anorexia, hypertension, ulcerative colitis or as anti-inflammatory, antiemetic, diaphoretic, antispasmodic and analgesic agents (*4*).

To date, only the volatile composition of mint honey has been analyzed, without detailed analysis of the broader bioactive composition or phenolic profiling (*5*). We performed pollen analysis, determined the basic physicochemical parameters, total phenolic and flavonoid contents, and antioxidant potential *via* two different methods: DPPH (2,2-diphenyl-1-picryl-hydrazyl-hydrate) free radical and ABTS (2,2’-azinobis (3-ethylbenzthiazoline-6-acid)) assays. Additionally, we analyzed the phenolic composition of these mint honeys *via* both qualitative and quantitative liquid chromatography coupled tandem mass spectrometry (LC-MS/MS) (*6*).

## MATERIALS AND METHODS

### Samples

**Fig. 1:**
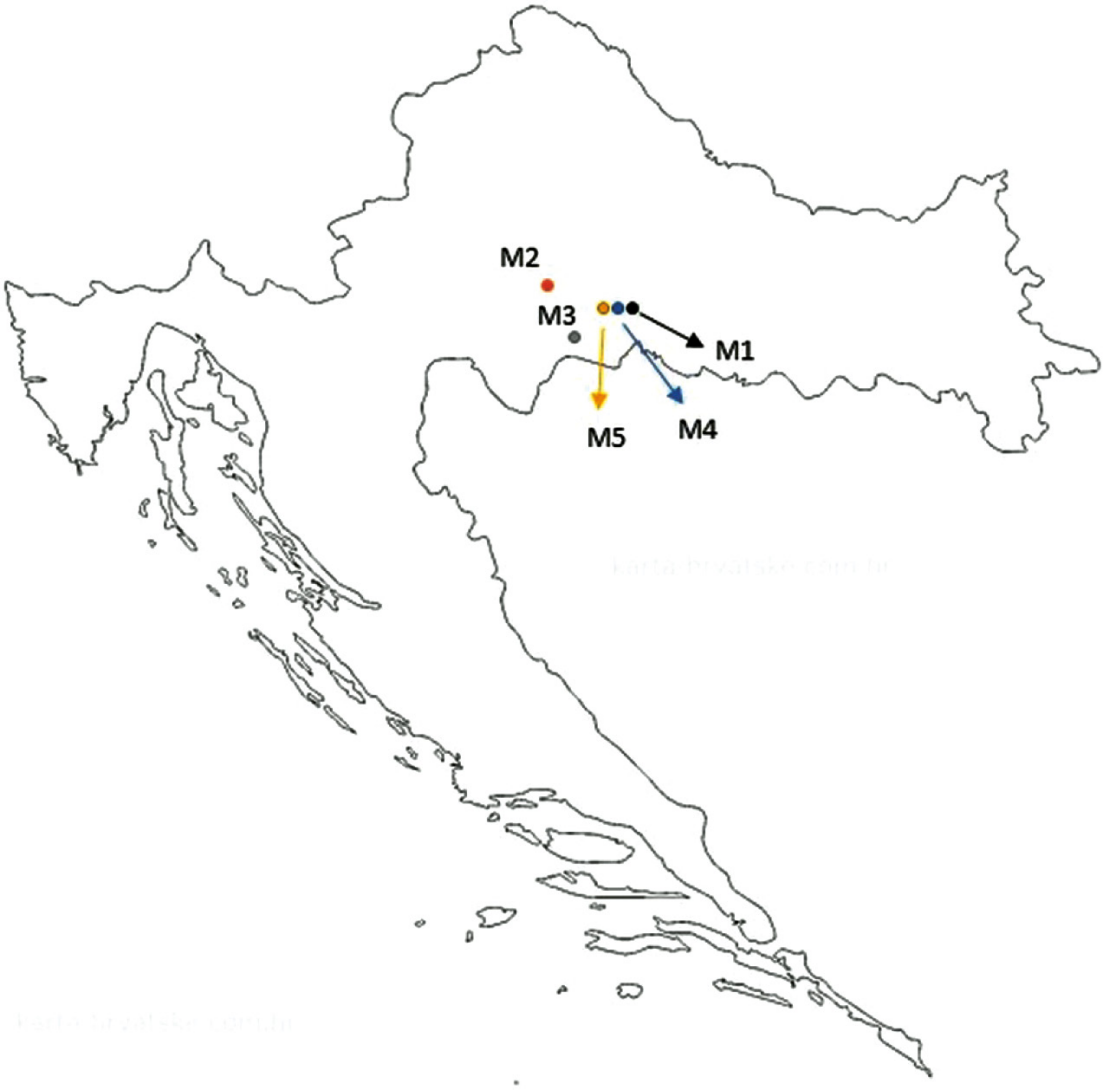
Locations of mint (*Mentha* spp.) honey producers in Croatia that provided samples for the study

Five samples of mint (*Mentha* spp.) honey were collected directly from producers at different geographical locations in Croatia (Fig. 1). M3 sample was produced in 2019, while the remaining four samples were produced in 2020.

### Reagents and materials

Phenolic standards: 2,5-DHBA, 3,4-DHBA, apigenin, caffeic acid, chrysin, kaempferol, luteolin, myricetin, naringenin, *p*-coumaric acid and quercetin were obtained from Cayman Chemical Company (Ann Arbor, MI, USA). Ethanol (LC-MS purity) was purchased from Sigma-Aldrich, Merck (St. Louis, MO, USA). Methanol (LC-MS purity) and acetonitrile (ACN; LC-MS purity) were purchased from VWR Chemicals BDH® (Radnor, PA, USA). The 2,2-diphenyl-1-picrylhydrazyl (DPPH) and 2,2'-azino-bis (3-ethylbenzothiazoline-6-sulfonic acid (ABTS)) were purchased from Alfa Aesar (Haverhill, MA, USA). The (±)-6-hydroxy-2,5,7,8-tetramethyl-chroman-2-carboxylic acid (Trolox) was purchased from Sigma-Aldrich, Merck. Aluminium chloride 6-hydrate was purchased from Gram-Mol (Zagreb, Croatia).

Milli-Q water was obtained using connected Ultrapure Water Systems (GenPure UV-TOC/UF xCAD plus) and Milli-Q water purification system (<0.055 μS/cm, Milli-Q Model Pacific TII 12; Thermo Fisher Scientific, Waltham, MA, USA).

### Standard physicochemical parameters

Standard physicochemical parameters determined for the mint honey samples were as follows: water content, electrical conductivity, pH, free acidity, hydroxymethylfurfural (HMF) content, apparent reducing sugars and apparent sucrose content. All methods were performed according to the International Honey Commission (IHC) as follows.

Water content was determined by digital refractometer PAL22S (Atago, Tokyo, Japan) according to the method: ’Determination of water with digital and Abbe refractometers’ (*7*). Briefly, honey was dissolved in a heating bath at 50 °C. Refractive index was measured at 20 °C after waiting for 6 min for equilibration.

The electrical conductivity of honey was determined according to the method: ’Determination of electrical conductivity’ (*8*). A mass of 20 g of honey was dissolved in 100 mL distilled water at 20 °C. Electrical conductivity cell was used for measurement.

The pH value was determined according to the method: ’Determination of pH and free acidity by titration to pH=8.3’ (*9*). For pH measurement pH meter (Mettler Tolledo, Columbus, OH, USA) was used.

Free acidity was determined according to the official IHC method: ’Determination of pH and free acidity” (*9*). A mass of 10 g of honey sample was dissolved in 75 mL of distilled water. Afterwards, the solution was titrated with 0.1 M sodium hydroxide to pH=8.30.

The HMF content was evaluated using HPLC/DAD system Agilent 1200 (Agilent Technologies, Santa Clara, CA, USA) according to the method for determination of hydroxymethylfurfural by HPLC (*10*). For mobile phase, *V*(water):*V*(methanol)=9:1 was used, flow rate was 1.0 mL/min and injection volume was 20 µL. A mass of 10 g of honey samples was dissolved in 50-mL beaker. The HMF content of the sample was calculated by comparing the corresponding peak areas of the sample and those of the standard solutions.

Apparent reducing sugars were quantified as previously described (*11*). Briefly, Fehling I solution was prepared by dissolving 69.28 g of copper sulfate pentahydrate in 1000 mL of distilled water and Fehling II solution was prepared by dissolving 346 g sodium potassium tartrate and 100 g sodium hydroxide in 1000 mL of distilled water. Apparent reducing sugars (g invert sugar per 100 g honey) were calculated *via* titration using methylene blue as an internal indicator and a pure sucrose solution as a standard.

Apparent sucrose content was determined by calculating the difference in total reducing sugar before and after complete inversion *via* acid hydrolysis (*11*). The difference in mass fractions of invert sugars was multiplied by 0.95 and expressed as g apparent sucrose per 100 g honey.

### Pollen analysis

The *Mentha* L. species were determined by the shape and size of the pollen grain and the structure and colour of the pollen outer wall (*12*). Briefly, 10 g of honey sample were dissolved in 20 mL of distilled water, heated in a water bath to 45 °C and centrifuged for 15 min at 1375×*g* (3500 rpm). The obtained sediment was used to prepare samples for microscopic analysis. Two parallel samples of the same honey were always made (*13*, *14*).

### Total phenolic content

A modified Folin-Ciocalteu method was used to determine total phenols (*15*). Briefly, 1 g of honey sample was dissolved in 10 mL of Milli-Q water and homogenized. A volume of 0.1 mL of the obtained solution was mixed with 0.1 mL Folin-Ciocalteu reagent and 0.9 mL Milli-Q water. After 5 min of incubation at room temperature, 0.8 mL of the 7.5% sodium carbonate solution was added and absorbance was measured at 760 nm after 20 min of incubation on a monochromator instrument Infinite M200 PRO (Tecan, Männedorf, Switzerland). Solutions of gallic acid in Milli-Q water in the concentration range of 0-200 mg/L were used to construct the calibration curve. The obtained results were expressed in mg of gallic acid equivalents (GAE) per 100 g honey.

### Total flavonoid content

A method according to Sousa *et al.* (*16*) was applied to determine total flavonoids. Briefly, honey samples were dissolved in 80% methanol to a concentration of 0.1 g/mL. The sample solutions were mixed with aluminium chloride methanol solution (2 g/100 mL) at a ratio of 1:1 (*V*/*V*). After 10 min of incubation at room temperature, absorbance was measured at 415 nm. Quercetin solutions in the concentration range 0-75 mg/L were used to construct the calibration curve. The obtained results were expressed in mg of quercetin equivalents (QE) per 100 g honey.

### ABTS method

A modified ABTS method of Sousa *et al.* (*16*) was used to study the antioxidant activity of honey). ABTS is a cationic radical formed by the reaction of 7 mM ABTS and 2.4 mM potassium persulfate, after allowing the mixture to stand in the dark for 14 h at room temperature. Samples were prepared by dissolving honey in methanol to a concentration of a 50 mg/mL. Series of Trolox concentrations ranging from 0 to 0.21 mM were used to construct the calibration curve. A volume of 40 μL of Trolox solution or sample solution was mixed with 160 mL of ABTS solution, which was previously diluted in methanol to an absorbance of 0.7. The absorbance values were measured ​​at 734 nm after 7 min of incubation using a monochromator instrument. The results were expressed in mg Trolox equivalents (TE) per 100 g honey.

### DPPH method

For the DPPH assay, honey samples were dissolved in methanol to a final concentration of 75 mg/mL. A solution of DPPH radical was prepared by dissolving DPPH in methanol at a concentration of 0.1 mM. A volume of 40 mL of the honey sample and 160 mL of the DPPH solution were mixed, and the absorbance was measured at 517 nm on a monochromator instrument after 60 min of incubation. A solution of Trolox in methanol at a concentration ranging from 0 to 0.21 mM was used to construct the calibration curve. The percentage of free radical inhibition was calculated and expressed in mg TE per 100 g honey.

### Solid phase extraction

Phenolic compounds were extracted using solid-phase extraction (SPE) performed according to the modified method of An *et. al.* (*17*), for which SPE polypropylene columns CHROMABOND® C18 ec, 6 mL, 500 mg, with polyethylene filter (Macherey-Nagel, Düren, Germany) were used. A mass of 1 g of honey was dissolved in 25 mL of Milli-Q water acidified with 0.1% formic acid (*V*/*V*) (pH=2.8) and homogenized. The C18 columns were connected manifold to a vacuum using a vacuum pump. SPE columns were conditioned with 3 mL of water, 3 mL of acidified Milli-Q water, and 3 mL of methanol. Solutions of the honey were then passed through the columns, followed by rinsing the sample with 5 mL of acidified water, 5 mL of Milli-Q and 5 mL of methanol. The methanol fraction was collected and filtered through a 25-mm 0.2 μm polytetrafluoroethylene filter (Agilent Technologies) and analyzed *via* LC-MS/MS.

### LC-MS/MS *analysis*

For quantitative and qualitative analyses of phenolic compounds in honey samples, Agilent 1260 series HPLC chromatograph (equipped with a degasser, binary pump and autosampler) and column oven coupled to Agilent 6460 triple quadrupole mass spectrometer (equipped with jet stream electrospray source) was used. For chromatographic separation of phenolic compounds, analyses were performed on Purospher STAR RP-18 Hibar HR column (50 mm×2.1 mm, 1.7 µm; Merck, Darmstadt, Germany). Herein we used a modified method from our previous work (*18*).

Briefly, phenolic standards were diluted in methanol at 15 different concentrations, after which calibration curves were generated and the linearity range was determined. Calibration curves were constructed using linear regression and were not forced to pass through zero. A 1/x statistical weight was applied in order to obtain the most reliable calibration curves. Linearity was determined using the coefficient of determination (R^2^). The limit of detection (LOD) and limit of quantification (LOQ) were calculated for each standard according to the guidelines of International Conference on Harmonization (ICH) (*19*). These parameters were calculated to select appropriate concentrations of standard mixtures for the standard addition method of quantification. Optimization parameters for quantitative LC-MS/MS analysis are given in Table S1.

For qualitative analysis of honey extracts, precursor and product ion scan modes were used with the same MS parameters as described above. The fragmentor was set at 80 and/or 135 V and collision energies were adjusted (0−40 V) for each identified compound. Phenolic compounds were identified by comparing precursor and product ions with the data available in the literature and by using the following databases: MassBank (*20*), mzCloud (*21*) and ReSpect (*22*).

### Absolute quantification of phenolic compounds by using the standard addition method

When the standard addition method was used, a calibration curve was constructed for each phenolic compound by adding a series of different concentrations (0.1, 0.2 and 0.4 mg/L) of the target compound to each sample prior to SPE. In this way, the calibration curve was prepared in the same honey that was analyzed, which neutralized the negative interference of the matrix with the final results. Different concentrations of the target compound were added to each sample, whereupon increases in LC-MS/MS intensities were measured, resulting from an increase in the amount of analyte in the sample. Linear regression analysis was used on the result points (data) and the analyte concentration in the sample (x-axis) was determined by extrapolating the regression line assuming that y=0.

### Statistical data processing

Statistical data processing was performed in MassHunter Qualitative analysis v. B.07.00 (*23*). Analysis of variance (one-way ANOVA) was conducted using The Data Analysis Toolpak in Excel 2016 (*24*). The implementation of principal component analysis (PCA) in the Python library Scikit-learn v. 0.20.3 (*25*) was used.

## RESULTS AND DISCUSSION

Melissopalynology or pollen analysis of honey is an important factor in the quality control of unifloral bee products and is included in the national legislation (*26*). In addition to sensory and physicochemical analysis, it is used to determine and control the botanical and geographical origin of honey, together with the analysis of volatile components. Therefore, we first performed pollen analysis of the obtained mint (*Mentha* spp.) honey samples to confirm that they are unifloral. According to the rules on the quality of unifloral honey (*27*), mint honey can be labelled as unifloral honey if the insoluble sediment contains at least 20% pollen grains of *Mentha* species. Table 1 lists the results of pollen analysis for all samples. Among all samples, the highest *Mentha* spp. pollen content was observed in samples M2 and M5.

**Table 1:** Standard physicochemical parameters determined for the mint (*Mentha* spp.) honey samples

Sample	*w*(water)/%	*σ*/(mS/cm)	pH	*b*(free acid)/(mmol/1000 g)	*w*(HMF)/(mg/kg)	*w*(reducing sugar)/(g/100 g)	*w*(sucrose)/(g/100 g)	*w*(pollen)/%
M1	19.1	0.77	3.83	37	<1	68.77	2.53	23
M2	19.8	0.66	3.63	37	3.4	67.57	2.43	28
M3	16.8	0.75	3.95	35	9.1	67.11	4.59	21
M4	17.4	0.80	3.65	37	10.3	74.29	2.95	25
M5	19.6	0.80	3.55	38	6.8	70.52	2.35	28

*σ*=electrical conductivity

European Union Directive 2001/110/EC (*2*) set the thresholds for all analyzed standard physicochemical parameters, where water content should be below 20%, electrical conductivity below 0.80 mS/cm, free acidity below 50 mmol/kg, hydroxymethylfurfural (HMF) content below 40 mg/kg, reducing sugars above 60 and sucrose content below 5 g/100 g . All analyzed honey samples were in compliance with these standards (Table 1).

Mint honey samples showed high total phenolic content, expressed in gallic acid equivalents (GAE), ranging from (76.7±0.6) to (90.1±1.1) mg100 g (Table 2). Pauliuc *et al*. (*28*) reported significantly lower total phenolic content in peppermint (*Mentha piperita*) honey, with mean value, expressed as GAE, of (23.7±4.37) mg/100 g . However, they reported interestingly high total flavonoid content, expressed as quercetin equivalents (QE), of (25.7±10.55) mg/100 g, exceeding the total amount of phenols. Our results showed that mint honey flavonoid content, expressed as QE, ranged from (6.7±0.6) to (12.5±0.8) mg/100 g (Table 2). Our results of the DPPH antioxidant activity were not in line with Pauliuc *et al.* (*28*), who reported (74.03±5.84) % inhibition, while the inhibition in our samples ranged from (33.6±2.8) to (51.3±1.2) % (Table 2). Samples M2 and M5, with the highest mint content (28%), showed similar antioxidant activity when analyzed with DPPH assay, whereas ABTS assay showed some differences. This can be the result of different affinities of the assays against other compounds that are present in the honey. Antioxidant activity is also affected by components in the honey that come from other plant species and can be up to 80% in different proportions in honey samples. Therefore, it may happen that mint honey samples have similar antioxidant activity, but at the same time a large difference in the amount of mint pollen grains. In addition to the above, we must not neglect the large number of different *Mentha* species that also potentially affect the different ratios and types of bioactive components.

**Table 2:** Total phenolic content, total flavonoid content and antioxidant activity measured by DPPH and ABTS assays of mint (*Mentha* spp.) honey

Sample	Total phenols *w*(GAE)/(mg/100 g)	Total flavonoids *w*(QE)/(mg/100 g)	DPPH *w*(TE)/(mg/100 g)	ABTS *w*(TE)/(mg/100 g)
M1	89.8±0.2	11.6±0.4	40.9±1.9	38.4±0.6
M2	78.4±1.3	10.6±0.5	42.7±1.9	30.4±0.7
M3	90.1±1.1	12.5±0.8	51.3±1.2	44.8±1.9
M4	78.4±0.3	7.8±0.2	33.6±2.8	14.4±0.8
M5	76.7±0.6	6.7±0.6	36.7±2.0	55.1±2.4

Results are expressed as mean value±standard deviation (S.D.), *N*=3. The results of one-way ANOVA showed significant differences in all analyzed parameters (p<0.0001). Some samples vary greatly from others, so these results were removed from further analysis

**Fig. 2:**
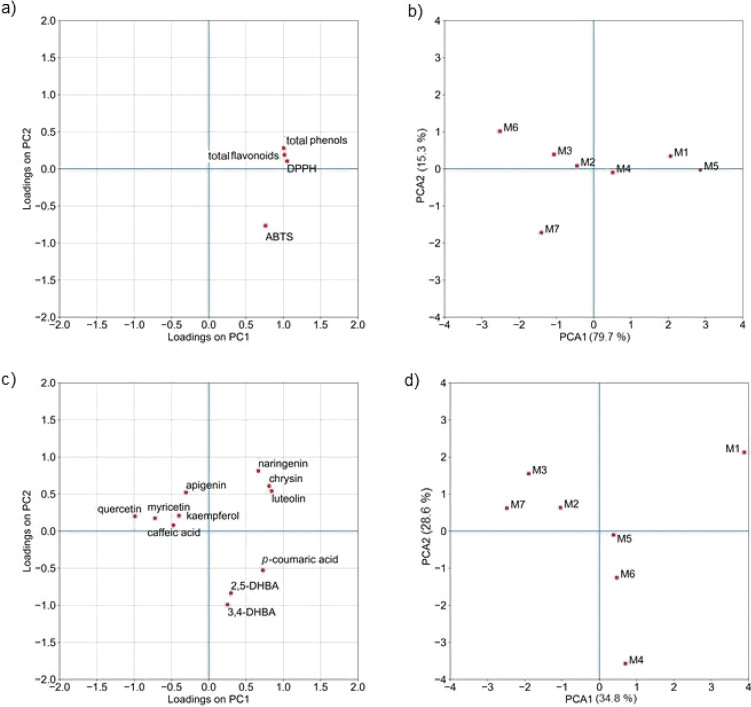
Distribution of elements (variables and samples respectively) in principal component 1 (PC1) *vs* principal component 2 (PC2) plot when: a and b) total phenolic content, total flavonoid content, DPPH and ABTS antioxidant activities, and c and d) quantified phenolic compounds (LC-MS/MS) in mint honey samples were used as variables

To test the variability among analyzed samples, we performed PCA analysis (Fig 2). The PCA projection of the total phenolic content, total flavonoid content, DPPH and ABTS antioxidant activities (Figs. 2a and 2b) indicated a high degree of heterogeneity of these variables among mint honey samples. The first two components (PC1 and PC2) describe 91.7% of total variability. Analyzed parameters enabled grouping of samples M1 and M3 on the positive side of PC1, while the rest of the samples were spread on the negative side. These samples had the lowest mint pollen content and antioxidant capacity, but the highest phenolic content. Therefore, it could be presumed that the presence of mint pollen results in honey with high content of phenols, but low antioxidant capacity, meaning that mint honeys have other components with antioxidant power. Since mint honey can contain as little as 20% of its pollen, it raises a question from what species are other honey components that also influence the analyzed parameters. Indeed, the total variability among all analyzed samples may result from the presence of components derived from plants other than *Mentha* species, or from the presence of different *Mentha* species.

To get a deeper insight into the antioxidant content of the mint honey, we performed a detailed quantitative LC-MS/MS analysis of major phenolic compounds. A major challenge for quantitative analysis of individual phenolic compounds in honey is the matrix effect, which is difficult to overcome given the lack of commercially available isotope-labelled internal standards. Due to its construction, LC-MS/MS analysis is sensitive to the matrix effect, which can affect the sensitivity and selectivity, reducing the accuracy, precision and robustness of the method itself. For this reason, we used the standard addition method. This method effectively compensates for low recovery and matrix effects. It is suitable for use when the matrix effect is strong, and no matrix standard is available to construct the calibration curve (external matrix matched calibration).

Quantitative analysis of phenolic compounds revealed that the most abundant flavonoids in mint honey are chrysin and apigenin at mass fractions ranging from (0.112±0.005) to (0.51±0.07) mg/100 g and from (0.1065±0.0002) to (0.64±0.01) mg/100 g, respectively (Table 3). Among the analyzed phenolic acids, *p*-coumaric acid was the most abundant with mass fractions up to (0.8±0.2) mg/100g. Pauliuc *et al*. (*28*) reported similar results for *p*-coumaric acid in honey of (0.61±0.53) mg/100 g. In addition, similar results were observed for caffeic acid and quercetin, while we observed significantly lower myricetin mass fraction ((0.0004±0.0004)–(0.0058±0.0001) mg/100 g). However, in our method a strong matrix effect of honey was observed, especially for myricetin, which had a falsely high signal in the MS spectrum and consequently a falsely high concentration. Therefore, these results should not be compared to those obtained using the relative quantification method. This indicates the advantage of using internal standards or the standard addition method.

**Table 3:** Mass fractions of specific phenolic acids and flavonoids in mint (*Mentha* spp.) honey samples obtained by LC-QQQ method

Phenol	*w*/(mg/100 g)					
M1		M2	M3	M4	M5
2,5-DHBA		<LOQ	<LOQ	0.045±0.008	0.014±0.002	0.0133±0.0008
3,4-DHBA		0.178±0.001	0.07230.002	0.6±0.2	0.3±0.1	NA
Apigenin		0.24±0.02	0.64±0.01	0.109±0.001	0.18±0.01	0.1065±0.0002
Caffeic acid		0.191±0.008	0.13±0.02	0.15±0.05	0.115±0.009	0.277±0.208
Chrysin		0.5±0.1	0.29±0.02	0.112±0.005	0.51±0.07	0.2506±0.0073
Kaempferol		0.059±0.002	0.072±0.003	0.0337±0.0005	0.07±0.02	0.0348±0.0003
Luteolin		0.0164±0.0003	0.0108±0.0002	0.0142±0.0004	0.0096±0.0002	0.018±0.001
Myricetin		0.0042±0.0001	0.002±0.002	0.0017±0.0005	0.0004±0.0004	0.0058±0.0001
Naringenin		0.0314±0.0002	0.0315±0.0000	0.01±0.02	0.0254±0.0002	0.0255±0.0006
*p*-coumaric acid		0.53±0.04	0.37±0.02	0.7±0.2	0.8±0.2	0.34±0.02
Quercetin		0.1015±0.0006	0.1279±0.0001	0.0580±0.0004	0.0960±0.0004	0.15±0.02

Results are expressed as mean value±standard deviation (S.D.), *N*=2. LOQ=limit of quantification, DHBA=dihydroxybenzoic acid, NA=not applicable

Figs. 2c and 2d show the PCA projection of quantitative LC-MS/MS analysis of phenolic compounds where all analyzed phenolic compounds were used as variables. The first two components describe 85.3% of the total variability. This high variability among all mint honey samples indicates that there are some differences that may be attributed to the variability in the individual *Mentha* spp. or the presence of additional plant species in the honey samples that may affect phenolic composition.

Finally, to detect the presence of other phenols in mint honey samples that were not included in the quantitative analysis, we performed MS screening with the same LC-MS/MS instrument. Results of this qualitative analysis are shown in Table 4. In total, 21 compounds were detected in all samples, among which 18 were identified. The *m/z* values of unidentified compounds that were present in all honey samples were 550.9(-), 553.0(-) and 771.2(+). Since there are no previous reports of phenolic profiling of mint honey, we compared our findings with the phenolic profiles of different plant parts of *Mentha* species. Mimica-Dukic and Bozin (*4*) described a large number of flavonoids and glycosides in *Mentha* species. Similar results were observed by Cirlini *et al.* (*29*) in their study of *Mentha spicata* (L.) extracts, who identified several salvianolic acids, rosmarinic acid derivatives and a large number of flavonoid glycosides. In our mint honey samples, we identified luteolin glycoside in M1, M2 and M5, and quercetin rutinoside in M1. Among the flavonoid aglycones, we found pinobanksin, diosmetin, galangin, apigenin, chrysin, kaempferol, luteolin, myricetin, naringenin and quercetin to be present in all samples (Table 3 and Table 4). The phenolic profiles of Tunisian native *Mentha rotundifolia* L. Huds were confirmed to contain 17 different phenolic compounds, among which salvianolic acids were most prominent (*30*). Interestingly, in our mint honey samples, we detected salvianolic acid G only in sample M2. Ćavar Zeljković *et al.* (*31*) reviewed the most abundant phenolic components in different types of *Mentha* species and at different geographical locations in Europe. They reported that the dominant phenolic compounds in *Mentha longifolia* species in Croatia are rosmarinic and chlorogenic acids. Indeed, we confirmed the presence of chlorogenic acid in four out of five mint honeys. Along with chlorogenic acid, we also observed the presence of caffeic acid and luteolin in all samples, which are usually found in the *Mentha aquatica* and *Mentha spicata* species (*31*). In addition, lithospermic acid, a compound specific for *Mentha longifolia*, *Mentha* × *piperita* and *Mentha* × *villosa* species, was detected in samples M1, M4 and M5.

**Table 4:** Identified compounds in mint (*Mentha* spp.) honey by LC-MS/MS method

Tentative identification	*t*_R_	Precursor mass (*m/z*)	Fragmentation pattern (*m/z*)	Molecular formula	Presence in analyzed honey samples					Level of confirmation*
M1	M2	M3	M4	M5
Salvianolic acid G	4.5	417.0 (-)	219.0 [C_11_H_7_O_5_]^-^ 237.0 [C_11_H_9_O_6_]^-^	C_20_H_18_O_10_	-	+	-	-	-	2
Unknown	4.6	354.0 (-)	279.1 264.8 235.0	/	+	-	+	+	+	3
Agathadiol	4.9	307.0 (+)	271.0 243.0 231.0 215.0 193.0 173.0	C_20_H_34_O_2_	+	+	+	+	+	2
*p*HBA	5.2	136.8 (-)	92.7 [C_6_H_5_O]^-^ 64.8 [C_4_HO]^-^	C_7_H_6_O_3_	+	+	+	+	+	1
2,5-DHBA (gentisic acid)	5.2	152.8 (-)	108.8 [C_6_H_5_O_2_]^-^ 52.9 [C_3_HO]^-^	C_7_H_6_O_4_	+	+	+	+	+	1
Unknown	5.3	667.0 (-)	343.0 322.7 178.8	/	+	-	-	+	+	3
Unknown	5.3	550.9 (-)	504.9 322.7 178.8	/	+	+	+	+	+	3
Unknown	5.4	553.0 (-)	506.9 322.9 178.8	/	+	+	+	+	+	3
Dimethyl caffeic acid	5.8	209.0 (-)	190.7 [C_10_H_7_O_4_]^-^ 162.9 [C_10_H_11_O_2_]^-^	C_11_H_12_O_4_	-	-	-	-	+	2
Chlorogenic acid	5.9	359.0 (-)	191.0 [C_7_H_10_O_6_]^-^	C_16_H_18_O_9_	+	-	+	+	+	1
Unknown	6	388.9 (-)	253.1 223.0 180.9	/	-	+	+	+	+	3
Rosmarinic acid	6	359.0 (-)	197.0 [C_9_H_9_O_5_]^-^ 179.0 [C_9_H_7_O_4_]^-^ 161.0 [C_9_H_5_O_3_]^-^ 135.0 [C_7_H_3_O_3_]^-^ 121.0 [C_7_H_5_O_2_]^-^	C_18_H_16_O_8_	-	-	+	-	-	2
Isocupressic acid	6.1	321.0 (+)	257.0 247.0	C_20_H_32_O_3_	-	-	+	-	-	2
Quercetin rutinoside	6.5	609.0 (-)	301.0 [C_15_H_7_O_7_]^-^ 271.0 [C_14_H_7_O_6_]^-^ 151.0 [C_7_H_3_O_4_]^-^	C_27_H_10_O_16_	+	-	-	-	-	2
Syringic acid	6.7	197.0 (-)	181.8 166.9 122.6	C_9_H_10_O_5_	+	+	+	+	+	1
Lithospermic acid	7	537.0 (-)	493.0 356.0 295.0	C_27_H_22_O_12_	+	-	-	+	+	2
Ferulic acid	7.2	192.9 (-)	177.9 [M-H_2_O]^-^ 149.0 [C_9_H_9_O_2_]^-^ 134.0 [C_8_H_4_O_2_]^-^	C_10_H_10_O_4_	+	+	+	+	+	1
Luteolin glucoside	7.3	449.1 (+)	287.0 [C_15_H_10_O_6_]^-^	C_21_H_20_O_11_	+	+	-	-	+	1
Narirutin (naringenin rutinoside)	7.5	581.0 (+)	545.0 [C_27_H_29_O_12_]^+^ 435.0 [C_21_H_23_O_10_]^+^ 419.0 315.0 273.0 [C_15_H_13_O_5_]^+^	C_27_H_32_O_14_	-	+	-	-	-	2
Unknown	7.7	771.2 (+)	609.0 462.9 600.6	/	+	+	+	+	+	3
Azelaic acid	8.4	287.0 (-)	125.0 [C_8_H_13_O]^-^	C_9_H_16_O_4_	+	-	-	-	+	2
*p*-coumaric prenyl ester	8.5	233.0 (-)	146.0 [C_9_H_5_O_2_]^-^ 118.8 [C_9_H_7_O_3_-CO_2_]^-^ 92.9	C_14_H_16_O_3_	+	+	-	+	+	2
Quercitrin	8.5	447.0 (-)	301.0 [C_15_H_10_O_7_]^-^ 300.0 [C_15_H_9_O_7_]^-^ 271.0 [C_14_H_7_O_6_]^-^ 151.0 [C_7_H_3_O_4_]^-^	C_21_H_20_O_11_	+	-	-	+	+	2
Dimethyl caffeic acid	8.6	209.0 (+)	190.7 [C_11_H_11_O_3_]^+^ 162.9 [C_9_H_7_O_3_]^+^ 133.0 [C_9_H_9_O]^+^ 118.9 [C_7_H_3_O_2_]^+^	C_9_H_8_O_4_	+	+	+	+	+	2
Abscisic acid	8.9	263.0 (-)	219.1 [C_14_H_19_O_2_]^-^ 203.8 [C_13_H_15_O_2_]^-^ 152.9 [C_9_H_13_O_2_]^-^	C_15_H_20_O_4_	+	+	+	+	+	2
Sebacic acid	9.2	200.9 (-)	182.8 [C_10_H_15_O_3_]^-^ 138.8 [C_9_H_15_O]^-^ 110.9 [C_8_H_15_]^-^	C_10_H_18_O_4_	+	+	+	+	+	2
Pinobanksin methyl ether	9.6	285.0 (-)	252.9 [C_15_H_9_O_4_]^-^ 240.9 239.0 226.9 [C_14_H_11_O_3_]^-^ 223.9	C_16_H_14_O_5_	+	+	-	+	+	2
Sakuranetin	9.6	284.9 (-)	164.9 [C_8_H_5_O_4_]^–^ 136.0 118.9 [C_7_H_3_O_2_]^-^ 108.0 92.8 [C_6_H_5_O]^-^	C_16_H_14_O_5_	-	+	-	+	+	1
Quercetin methyl ether	10	315.0 (-)	300.0 [M-CH_3_]^–^ 270.7 [M-CH_3_-CO]^–^ 255.0	C_16_H_21_O_7_	+	+	+	+	+	2
Tectochrysin	10.5	267.0 (-)	252.0 224.0 [C_10_H_9_O_6_]^-^	C_16_H_12_O_4_	+	+	+	+	+	1
Pinobanksin	10.8	271.0 (-)	252.9 [C_15_H_9_O_4_]^-^ 225.0 [C_14_H_9_O_3_]^-^ 196.6 [C_13_H_9_O_2_]^-^ 160.7 [C_10_H_9_O_2_]^-^	C_15_H_12_O_5_	+	+	+	+	+	1
Diosmetin	10.9	301.0 (+)	285.9 [C_15_H_9_O_6_]^+^ 257.9 [C_14_H_9_O_5_]^+^ 228.7 [C_13_H_9_O_4_]^+^	C_16_H_12_O_6_	+	+	+	+	+	1
Kaempferide	11	299.0 (-)	284.0 [C_15_H_8_O_6_] 255.0 227.0 [C_13_H_7_O_4_]^-^	C_16_H_12_O_6_	+	+	+	+	+	2
Artepilin C	11	299.0 (-)	255.0 151.0	C_19_H_24_O_3_	+	-	-	+	+	2
Quercetin dimethyl ether	11	329.0 (-)	313.7 [M-CH_3_]^-^ 298.7 [M-2CH_3_]^-^ 270.8 [M-2CH_3_-CO]^-^	C_17_H_14_O_7_	-	+	+	+	+	2
Rhamnetin	11.3	314.9 (-)	165.0 [C_8_H_5_O_4_]^-^ 121.0	C_16_H_12_O_7_	-	+	+	--	+	1
Caffeic acid benzyl ether	11.4	269.0 (-)	178.0 [C_9_H_7_O_4_]^-^ 161.0 [C_9_H_5_O_3_]^-^ 134.0 [C_9_H_7_O_4_-CO_2_]^-^	C_16_H_14_O_4_	+	+	+	-	+	2
Caffeic acid prenyl ester (prenyl caffeate)	11.4	247.0 (-)	246.7 178.8 [C_9_H_7_O_4_]^-^ 160.8 [C_9_H_5_O_3_]^-^ 135.0 [C_9_H_7_O_4_-CO_2_]^-^	C_14_H_16_O_4_	+	+	+	+	+	2
Pinocembrin	11.5	254.8 (-)	212.9 [C_13_H_9_O_3_]^-^ 150.8 [C_7_H_3_O_4_]^-^	C_15_H_12_O_4_	+	+	+	-	+	1
Pinostrombin	11.5	269.0 (-)	167.1 [C_8_H_7_O_4_]^-^ 130.9 [C_9_H_7_O]^-^	C_16_H_14_O_4_	+	+	-	-	-	2
Pinobanksin-O-acetate	11.5	313.0 (-)	270.1 [C_15_H_12_O_5_]^-^ 252.9 [C_15_H_9_O_4_]^-^ 114.5	C_17_H_14_O_6_	-	-	-	+	+	2
Caffeic acid phenylethyl ether (CAPE)	11.5	283.0 (-)	179.0 [C_9_H_7_O_4_]^-^ 135.0 [C_9_H_7_O_4_-CO_2_]^-^	C_17_H_16_O_4_	+	+	+	+	+	2
Galangin	11.6	268.9 (-)	252.0 168.9 142.9	C_15_H_10_O_5_	+	+	+	+	+	1
Acacetin	11.7	283.0 (-)	268.0	C_16_H_12_O_5_	+	+	+	+	+	2
*p*-coumaric acid cinnamyl ester	12.5	281.0 (+)	241.0 192.6 162.1 147.0 [C_9_H_7_O_2_]^+^ 118.6 [C_8_H_7_O]^+^ 106.6	C_18_H_16_O_3_	+	+	+	+	+	2

*Level of confirmation: 1=standard, 2=literature or databases, 3=unknown. *p*HBA=*p*-hydroxybenzoic acid, DHBA=dihydroxybenzoic acid

The obtained results are the basis for further characterization of mint honey, which should include more samples from various geographical regions and containing pollen from different *Mentha* species. More detailed analysis (*e.g*. genetic analysis) of *Mentha* pollen in the honey samples may also be beneficial. Finally, additional comparative analysis of mint honey with other types of honey is necessary to confirm the presence of *Mentha*-specific honey constituents.

## CONCLUSIONS

In this paper, we conducted a detailed qualitative and quantitative analyses of phenols from mint (*Mentha* spp.) honey. In combination with the previously published results on volatile components, we have taken an additional step towards the full detailed characterization of this rare bee product. Here, we showed that mint honey has a unique phenolic fingerprint and high content of individual phenols. Indeed, spectrophotometric analysis showed high phenolic mass fraction, expressed as gallic acid equivalents, up to (90.1±1.1) mg/100 g that was accompanied by strong antioxidant activity measured with DPPH and ABTS methods. Quantitative LC-MS/MS analysis confirmed high mass fractions of chrysin, apigenin and quercetin, flavonoids with known strong biological activity. In addition, untargeted analysis indicates that mint honey can be a valuable source of rare phenolic compounds. Indeed, we confirmed the presence of several flavonoid glycosides (quercetin rutinoside, luteolin glucoside, narirutin, quercitrin, *etc*.), abscisic and sebacic acid, as well as the acacetin, galangin, pinostrombin and pinocembrin in all analyzed samples. Overall, our research has added valuable insight into the benefits of this rare bee honey and laid the foundation for greater product recognition in the food industry.

## Appendix

**Table S1:** MS/MS standard optimization parameters

Standard	Polarity	Precursor (*m/z*)	Fragmentor/V	Product ions (*m/z*)	CE/V	*t*_R_/min	a	b		R^2^	Linearity range/(µg/mL)	LOD/(µg/mL)	LOQ/(µg/mL)
2,5-DHBA	-	152.8	100	107.8*	20	5.20	26643.4743		-13.9153	0.9969	0.01-2.5	0.0017	0.0052
				52.9	20								
3,4-DHBA	-	152.8	90	108.8	10	4.24	28778.8402		2.1451	0.9954	0.01-2.5	0.0002	0.0007
				90.6	26								
				80.8	20								
Apigenin	-	268.7	160	224.5	20	10.72	4448.7078		5.0376	0.9974	0.01-1.0	0.0037	0.0113
				148.5	24								
				116.6	38								
Caffeic acid	-	178.8	80	137.7	12	5.79	187.5139		5.7729	0.9929	0.01-1.0	0.1016	0.3079
				106.4	24								
Chrysin	-	252.7	180	142.7	26	11.44	2319.0652		30.9715	0.9983	0.01-1.0	0.0441	0.1336
				118.6	30								
				106.5	24								
Kaempferol	-	284.7	190	238.5	28	10.86	1125.8622		7.0511	0.9951	0.01-1.0	0.0207	0.0626
				184.4	28								
				170.5	28								
Luteolin	-	284.7	200	242.8	20	9.45	30263.2753		68.3527	0.9962	0.01-1.0	0.0075	0.0226
				198.8	24								
				150.7	24								
				132.7	36								
Myricetin	-	316.7	160	270.7	26	8.19	6673.6056		727.8511	0.9927	0.01-2.5	0.3599	1.0906
				150.7	24								
Naringenin	-	270.8	120	150.6	14	10.60	24343.7076		428.4255	0.9903	0.01-0.5	0.0581	0.1760
				118.8	26								
*p*-coumaric acid	-	162.9	90	118.8	10	6.67	32493.0182		40.2011	0.9972	0.01-2.5	0.0041	0.0124
				92.6	34								
				65.7	44								
*p*HBA	-	136.8	80	92.7	12	5.18	6304.6523		9.7256	0.9922	0.01-1.0	0.0051	0.0154
Quercetin	-	300.8	170	178.5	14	9.50	7330.6106		-28.6147	0.9959	0.01-1.0	0.0129	0.0390
				150.6	20								

*Quantifier ions are underlined. DHBA=dihydroxybenzoic acid, *p*HBA=4-hydroxybenzoic acid, CE=collision energy, *t*_R_=retention time, a=slope and b=intercept (y=ax+b), LOD=limit of detection, LOQ=limit of quantification
